# Effect of Fermented Medicinal Plants as Dietary Additives on Food Preference and Fecal Microbial Quality in Dogs

**DOI:** 10.3390/ani9090690

**Published:** 2019-09-16

**Authors:** Da Hye Park, Damini Kothari, Kai-Min Niu, Sung Gu Han, Jee Eun Yoon, Hong-Gu Lee, Soo-Ki Kim

**Affiliations:** 1 Department of Animal Science and Technology, Konkuk University, Seoul 05029, Korea; eadg@naver.com (D.H.P.); damini.kth@gmail.com (D.K.); hglee66@konkuk.ac.kr (H.-G.L.); 2 Team of an Educational Program of Specialists in Global Animal Science, Brain Korea 21 Plus Project, Sanghuh College of Life Sciences, Konkuk University, Seoul 05029, Korea; 3 Institute of Biological Resource, Jiangxi Academy of Sciences, Nanchang 330029, China; niukaimin@naver.com; 4 Department of Food Science and Biotechnology of Animal Resource, Konkuk University, Seoul 05029, Korea; hansg@konkuk.ac.kr (S.G.H.); 7wlmds7@naver.com (J.E.Y.)

**Keywords:** antioxidants, dog foods, *Enterococcus faecium*, fermentation, food additives, food preference, medicinal plants

## Abstract

**Simple Summary:**

Dog foods are becoming more equivalent to human foods, and functional additives are being included in their diets to promote health. In this study, turmeric, glasswort, and Ganghwa mugwort were used as medicinal plants and were subjected to fermentation by autochthonous *Enterococcus faecium*. Fermentation significantly improved the *in vitro* antioxidant activities of these plants. Food preference tests of dog foods containing these fermented medicinal plants were conducted in beagles.

**Abstract:**

This research determined the antioxidant activities of medicinal plants fermented *by Enterococcus faecium* and their subsequent applications as dog food additives. Turmeric (5%, *w*/*v*), glasswort (2.5%, *w*/*v*), Ganghwa mugwort (2.5%, *w*/*v*), and their mixture (5%, *w*/*v*) were fermented by autochthonous *E. faecium* (1%, *v*/*v*) for 72 h. Bacterial cell counts and pH were monitored during fermentation. Total polyphenol content (TPC), total flavonoid content (TFC), 2,2′-azino-bis (3-ethylbenzothiazoline-6-sulfonic acid) (ABTS) and 1,1-diphenyl-2-picrylhydrazyl (DPPH) radical scavenging activity, and intracellular superoxide scavenging activity in bovine mammary alveolar epithelial (MAC-T) cells were measured with the fermented and non-fermented samples. Only the antioxidant capacity of the mixture was increased after fermentation. However, intracellular superoxide level in MAC-T cells was significantly reduced after treatment with fermented plant samples (*p* < 0.001) as compared with that in non-fermented plants. Fermented plants were then sprayed at 1% (*v*/*w*) onto dog foods. TPC, TFC, ABTS radical scavenging activity, and DPPH radical scavenging activity of dog foods were significantly enhanced after the addition of fermented plants. Food preference testing was conducted using a two-pan method—control diet vs. four treatment diets—for 4 days for each additive diet, a total 16 days in 9 beagles. Feces were collected to enumerate bacterial counts. Preferences for glasswort and Ganghwa mugwort were higher than those of the control (*p* < 0.05). Furthermore, fecal microbiota enumeration displayed a higher number of beneficial microorganisms in treated groups. These results suggest that fermented plants with enhanced antioxidant abilities might be useful as potential additives for dog foods.

## 1. Introduction

More and more people are paying attention to the health and welfare of their companion animals, especially dogs [1]. Dog owners’ interest in foods with known functional benefits is therefore becoming increasingly popular. Recently, antioxidants have emerged as important functional ingredients in the foods of companion animals due to their alleged benefits in human foods [2,3]. Antioxidants counteract the effects of reactive oxygen species or free radicals, generated by normal metabolism. Inefficient clean-up or accumulation of free radicals damages proteins, lipids, and nucleotides as well as suppresses the immune system, which in turn results in increased incidence of diseases like Alzheimer’s, Parkinson’s, multiple sclerosis, autoimmune disease, etc., within the senior human population. Similarly, dogs also show age-related maladies [4,5]. Milgram et al. [6] hypothesized that the short-term administration of an antioxidant-fortified food might alleviate/delay aging-associated adverse effects in dogs. Generally, pet manufacturers use synthetic antioxidants due to their efficacy, good carry through, and cost-effectiveness. However, concern about the toxicity of synthetic antioxidants intensified the hunt for natural antioxidants in pet foods [7]. In this context, dietary supplementation of plants with high antioxidant and free-radical scavenging capacities could be a safe and cost-effective strategy. In addition, plant extracts might enhance the flavor and shelf-life of dog food [8]. Among many medicinal plants, turmeric (*Curcuma longa*), glasswort (*Salicornia herbacea*), and Ganghwa mugwort (*Artemisia princeps*) were selected in this study, which are known to have abundant bioactive compounds with antioxidant effects.

Turmeric, produced from the rhizome of *Curcuma longa* L., is commonly used as a spice, food preservative, and coloring agent for thousands of years in India [9,10]. Korea also has a long history of the cultivation and medicinal use of turmeric for human health benefits. The phenolic curcuminoids are the major bioactive components of turmeric, responsible for its antioxidant, anti-inflammatory, and antimicrobial effects [10]. Sechi et al. [5] proposed a diet enriched with a mixture of plant-derived antioxidants (including turmeric as a component) and omega 3/6 fatty acids as a valid alternative and a valuable strategy to counteract aging-related cognitive decline in elderly dogs. Recently, the addition of curcumin to dog food was reported to more effectively reduce lipid oxidation as compared with the synthetic antioxidant butylated hydroxyanisole [8].

Glasswort (*Salicornia herbacea* L.) is an edible halophyte that grows along the high-salt coastal marshes of East Asia. It is commonly consumed as a raw vegetable or as a nutritious fermented food in Korea and European countries [11,12]. Glasswort contains high amounts of NaCl (3.40–20.19%) and other minerals such as calcium, magnesium, and iodine [13,14]. Additionally, glasswort contains several bioactive compounds, such as caffeic acid, trans-ferulic acid, acanthoside B, isorhamnetin, irilin B, protocatechuic acid, p-coumaric acid, and quercetin, which contributed to its antioxidant, antimicrobial, anticancer, lipid-lowering, and immunomodulatory activities [11,12]. Karthivashan et al. [11] reported the ameliorative action of glasswort ethanol extract on oxidative stress in mice. The antioxidative effect of 10% glasswort was similar to that of 1% α–tocopherol [15].

The perennial herb Ganghwa mugwort (*Artemisia princeps* Pampanini cv. Sajabal) is widely distributed in East Asia. The leaves are commonly used as tea and food and have also been used in traditional Asian medicine for the treatment of inflammation, diarrhea, gastric ulcer, bacterial infections, and circulatory disorders [16,17]. The leaves are rich in flavonoids (eupatilin, eupafolin, apigenin, and jaceosidin) as well as phenolic acids (caffeoylquinic acids) and are reported to have high antioxidant activity [17,18,19].

Food fermentation provides palatability, nutritional value, as well as preservative and medicinal properties [20]. Beneficial lactic acid bacteria (LAB) associated with fermented foods can improve the intestinal environment, strengthen the immune system, increase nutrient utilization, reduce lactose intolerance, and reduce specific cancer risk [21]. To our knowledge, no studies have been conducted on the fermentation of medicinal plants for their application as additives in dog foods. Herein, we hypothesize that fermentation will enhance the specific nutrient or phytonutrient content of foods and may act as a source of probiotics for dogs. Considering this background, the present study involves the fermentation of the above-mentioned medicinal plants and the subsequent addition to extruded dog foods in order to evaluate their potential application as functional ingredients. We also studied dog food preference and the effect of fermented plants on digestion by determining food intake and fecal microbial characteristics, respectively.

## 2. Materials and Methods

### 2.1. Isolation, Identification, and Selection of Bacterial Strains for Fermentation

Powders of turmeric (*C. longa*), glasswort (*S. herbacea*), and Ganghwa mugwort (*A. princeps* Pampanini cv. Sajabal) were purchased from Korean food companies (turmeric: Malg-eundeul Co., Hongcheon-gun Gangwondo, Korea; glasswort: Dasarang Agricultural Co., Sinan-gun, Jeollanamdo, Korea; Ganghwa mugwort: San-aedeul-ae Co., Ganghwa-gun, Incheon, Korea). Each plant (5 g) was added into 45 mL of sterilized CHO buffer (K_2_HPO_4_, NaCl, MgSO_4_, sodium acetate) [22], adjusted to pH 7.0 ± 0.5, and incubated in a shaking incubator (135 rpm) at 37 °C for 3 days. After 24 h of incubation, naturally inhabited microorganisms were isolated using nutrient agar (NA) (Difco Laboratories, Detroit, MI, USA), Reasoner’s 2A (R2A) agar (Difco Laboratories, Detroit, MI, USA), and de Man, Rogosa, and Sharpe (MRS) agar (Difco Laboratories, Detroit, MI, USA). Isolated microbial strains were purified by several trans-generations using corresponding isolated medium and identified by 16S rDNA sequence using a commercial service (Macrogen Inc., Seoul, South Korea).

### 2.2. Hemolysis and Antibiotic Resistance of Enterococcus faecium

The safety aspects of selected *E. faecium* strains (SK4357, SK4369, SK4373) in relation to hemolysis and antibiotic resistance were investigated. The hemolysis test was determined by the color change of blood agar (HiMedia Laboratories Pvt. Ltd., Mumbai, India) plates containing 5% (*v*/*v*) horse blood (Hanil komed Co. Ltd., Seongnamsi, Gyeonggido, Korea) [23]. Overnight *E. faecium* cultures were streaked onto blood agar plates and incubated at 37 °C for 48 h.

The antibiotic resistance of *E. faecium* was determined using the Kirby–Bauer disc diffusion method [24]. The antibiotics used in this study were cefepime (30 μg), gentamicin (2 μg), vancomycin (30 μg), ampicillin (10 μg), tetracycline (30 μg), oxacillin (1 μg), ciprofloxacin (5 μg), chloramphenicol (30 μg), and clindamycin (2 μg) (Oxoid Ltd., Basingstoke, Hampshire, UK). Overnight *E. faecium* cultures were swabbed onto MRS agar plates, and paper discs (8 mm diameter) containing antibiotics were then placed on each plate. After incubation (24 h and 37 °C), bacterial strains were evaluated as resistant or sensitive by measuring inhibition zone diameters around the antibiotic discs.

### 2.3. Fermentation of Medicinal Plants

The medicinal plant powders, 5% (*w*/*v*) turmeric, 2.5% (*w*/*v*) glasswort, 2.5% (*w*/*v*) Ganghwa mugwort, and 5% (*w*/*v*) mixture (1.66% (*w*/*v*) turmeric, 1.66% (*w*/*v*) glasswort, and 1.66% (*w*/*v*) Ganghwa mugwort) were added separately to *Bacillus* minimal media (BMM) (pH 7.0 ± 0.5) and the media was sterilized at 121 °C for 15 min. Then, *E. faecium* isolated from the corresponding medicinal plants was inoculated into the BMM containing respective plants and fermented for 72 h at 37 °C with shaking (135 rpm). Samples were collected at 0, 4, 8, 12, 16, 24, 48, and 72 h to determine bacterial counts and pH. The fermentation solution was collected at each time point and stored at −20 °C. The solution was thawed at room temperature (25 °C), filtered through Whatman No. 1 filter paper, and then used for analysis.

### 2.4. Preparation of Plant Extracts

After fermentation, filtered samples were centrifuged at 5000 rpm for 5 min. The collected supernatant (0.1 mL) was mixed with 0.3 mL of 80% (*v*/*v*) methanol and sonicated (60 Hz) for 10 min at 30 °C. Samples were centrifuged at 14,500 rpm for 10 min and supernatant was collected and stored at −20 °C for total polyphenol content (TPC), total flavonoid content (TFC), 2,2’-azino-bis (3-ethylbenzothiazoline-6-sulfonic acid) (ABTS) and 1,1-diphenyl-2-picrylhydrazyl (DPPH) analyses.

### 2.5. Estimation of Total Phenolic Content

The total phenolic contents of fermented and non-fermented samples were measured according to the Folin–Ciocalteu method [25]. Briefly, in a 96-well-plate, 20 μL sample and 100 μL 0.2 N Folin–Ciocalteu reagent was added. After 5 min of incubation, 80 μL 7.5% Na_2_CO_3_ in deionized water was added. After 1 h of incubation at room temperature in the dark, absorbance was measured at 765 nm using an ELISA reader (Synergy 2, BioTek Instruments Inc., Winooski, VT, USA). The absorbance was used to calculate total phenolic content based on a gallic acid standard curve. Results are expressed as gallic acid equivalent (GE μg/mL).

### 2.6. Estimation of Total Flavonoid Content

Total flavonoid contents of fermented and non-fermented samples were estimated according to a published method [26]. Briefly, in a 96-well-plate, 20 μL of sample, 180 μL of 90% diethylene glycol, and 20 μL of 1 N NaOH were added into each well and incubated in the dark at room temperature for 1 h. The absorbance was then measured at 405 nm using an ELISA reader (Synergy 2, BioTek Instruments Inc.). Quercetin was used as standard. Total flavonoid content was expressed as quercetin equivalent (QE μg/mL).

### 2.7. Determination of DPPH Radical Scavenging Activity

Antioxidant activities of plants were measured with the DPPH radical assay [27]. Briefly, in a 96-well-plate, 20 μL of sample and 180 μL of 0.15 mM DPPH solution were added. After incubation at room temperature in the dark for 30 min, absorbance of the sample was measured at 517 nm using an ELISA reader (Synergy 2, BioTek Instruments Inc.). The scavenging effect of DPPH radical by the samples was calculated according to the following Equation (1):

Radical scavenging activity (%) = (1 − Sample Absorbance/Control Absorbance) × 100%(1)

### 2.8. Determination of ABTS Radical Scavenging Activity

The antioxidant activities of plants were measured by ABTS radical assay [28]. The ABTS^+^ solution was made by mixing ABTS (2,2’-azino-bis (3-ethylbenzothiazoline-6-sulfonic acid)) (Sigma-Aldrich, St. Louis, MO, USA) solution (14.8 mM) with 5 mM potassium persulfate (1:1, *v*/*v*) and allowed to react at room temperature in the dark for 16 h. The ABTS^+^ solution was diluted with distilled water until the absorbance at 734 nm was 0.700 ± 0.05 before use. In a 96-well plate, 20 μL of sample and 180 μL ABTS^+^ solution were added. After incubation in dark at room temperature for 30 min, the sample absorbance was measured at 734 nm using an ELISA reader (Synergy 2, BioTek Instruments Inc.). The scavenging effect of ABTS radical by the samples was calculated according to Equation (1).

### 2.9. Assessment of Intracellular Superoxide Levels

Intracellular superoxide level was measured as described previously [29]. Bovine mammary epithelial cells (MAC-T) were grown until 90% confluency on a cover glass in a six-well plate. These MAC-T cells were treated with 50 μL of different fermented and non-fermented samples or dimethyl sulfoxide (DMSO) for 12 h and then treated with 1 μg/mL of lipopolysaccharide (LPS) for 4 h. Cells treated with DMSO were used as control. Treated and control cells were stained with 1 µM dihydroethidium (DHE) (Invitrogen, Woonsocket, RI, USA) for 30 min, followed by three PBS washes. After that, cells on coverslips were fixed with 4% formaldehyde, incubated for another 10 min, and washed three times with PBS. Nuclei of cells were stained with ProLong Gold Antifade reagent containing 4,6-diamidino-2-phenylindole (DAPI) (Life Technologies Inc., Gaithersburg, MD, USA). Lastly, cells were visualized with an Olympus IX71 fluorescence microscope at 200× magnification. Images were taken using an Olympus DP71 camera with DP controller software (Olympus Optical Co. Ltd., Tokyo, Japan). Intracellular superoxide levels were calculated according to the following Equation (2):

Intracellular superoxide level (%) = (Sample fluorescence intensity/Control fluorescence intensity) × 100%(2)

### 2.10. Preparation of Dog Foods

The basal dog foods were produced and supplied by AT Bio Co. (Pocheon-si, Gyeonggi-do, South Korea). Production was achieved by extrusion using a single-screw extruder (Wenger Co., Ltd., Sabetha, KS, USA) at the food rate of 1.5 Ton/h, screw rotation speed of 420 rpm, and with the barrel temperatures of four segments (120 °C, 4 gears; 120 °C, 5 gears; 115 °C, 6 gears; and 100 °C, 7 gears). The ingredients and nutrient composition of the basal dog food are shown in Table 1. The basal dog food was used as the control. The treatment diets were prepared by spraying the 16-h-fermented medicinal plants at 1% (*v*/*w*) concentration uniformly prior to the last oil coating step of extrusion. Then, control and treatment dog foods were dried at 40 °C for 10 h, coated with oil, crushed into pellets (6 mm × 6 mm × 1.5 mm; W × D × H), and kept at 4 °C until use.

### 2.11. Preparation of Dog Food Extracts and Analyses

The extraction was carried out by mixing 3 g of ground dog foods with 9 mL of 80% (*v*/*v*) methanol and sonicated (60 Hz) for 10 min at 30 °C. After sonication, samples were placed in a shaking (120 rpm) incubator at 30 °C for 16 h. Thereafter, extracts were centrifuged at 14,500 rpm for 10 min and supernatants were collected and stored at −20 °C until analyses. TPC, TFC, and antioxidant activities of dog food extracts were determined as described above.

### 2.12. Experimental Animals and Food Preference Test

The animal study was approved by the Konkuk University Institutional Animal Care and Use Committee (IACUC), Seoul, Republic of Korea, in accordance with the guidelines of IACUC (KU18157). Nine adult beagles of varying age (5–10 years) and weighing 12.5–14.5 kg were housed individually in indoor semi-open pens (700 mm × 700 mm × 690 mm; W × D × H) with controlled temperature (25 ± 2 °C) at the laboratory of Animal Research, Konkuk University, Korea. Prior to study, all dogs were given *ad-libitum* access to a different commercial diet (200–300 g/day) and water.

Food preference test was conducted using a two-pan method [30] with four diet comparisons: control vs. 1% fermented turmeric added diet, control vs. 1% fermented glasswort added diet, control vs. 1% fermented Ganghwa mugwort added diet, and control vs. 1% fermented mixture added diet. No food other than the experimental diets was provided during the study period. Every day at the same time between 16:00 h and 18:00 h, 500 g of treatment diet and 500 g of control diet were offered once in a pre-weighed bowl. Beagles were allowed to eat for 30 min only to avoid obesity [31]. After the feeding period, spilled food was collected and recorded with the remaining food in the bowl, and final weights were recorded. Each food preference test was run for four consecutive days by changing the direction of the left and right bowl every day to eliminate directional preference. The animals had access to an external environment (around the lab) for 30 min in between 13:00 and 15:00 h daily. All dogs had *ad-libitum* access to water. The beagles used in this study had previously experienced food preference testing. Food preference was determined by measuring food intake ratio. It was calculated using the following Equation (3):

Intake ratio = Consumed amount of A/(Consumed amount of A + Consumed amount of B)(3)

### 2.13. Fecal Bacteria Isolation and Counts

Fecal samples of six beagles were collected at the beginning (control) and at the end (after 4 days) of each diet preference test. Samples were immediately diluted and spread on LB and MRS agar plates for bacterial enumeration. Dominant and non-dominant microorganisms were isolated and identified by 16s rDNA sequencing by a commercial service, Macrogen Inc. (Seoul, Republic of Korea).

### 2.14. Statistical Analysis

All data are expressed as mean ± standard deviation (SD). All statistical analyses were performed using SPSS Version 22.0 software (IBM Corp., Armonk, NY, USA). Total polyphenol contents, total flavonoid contents, DPPH scavenging activity, intracellular superoxide scavenging activity, and food preference test were analyzed using Student’s *t*-test. All results with *p*-value < 0.05 were considered statistically significant.

## 3. Results

### 3.1. Isolation and Identification of the Strains from Medicinal Plants

A total of 19 microorganisms were isolated and identified in NA, R2A, and MRS agar plates (Table 2). In turmeric, Cronobacter sakazakii, Enterobacter aerogenes, Enterobacter cloacae, Enterococcus faecium, Enterococcus gallinarum, Klebsiella pneumoniae, Pediococcus pentosaceus, and Phytobacter diazotrophicus were isolated and identified. Enterococcus faecium, Enterococcus hirae, Weissella cibaria, and Bacillus nealsonii were identified from glasswort. Enterococcus faecium and Weissella cibaria were identified from Ganghwa mugwort. E. faecium was commonly isolated from turmeric, glasswort, and Ganghwa mugwort, and was therefore selected as the starter culture for the fermentation of these medicinal plants.

### 3.2. Hemolysis and Antibiotic Resistance

A clear zone in a blood agar (5% *v*/*v* horse blood) plate indicates β-hemolysis, and a green or brown color demonstrates α-hemolysis. No color change means γ-hemolysis (non-hemolytic activity). There was no color change in blood agar plates with *E. faecium* strains (SK4357, SK4369, SK4373), indicating their non-hemolytic nature (data not shown). Antibiotic susceptibility tests revealed that all these strains were sensitive to ampicillin, vancomycin, chloramphenicol, and ciprofloxacin (Table 3).

### 3.3. Fermentation Characteristics

**Figure 1:**
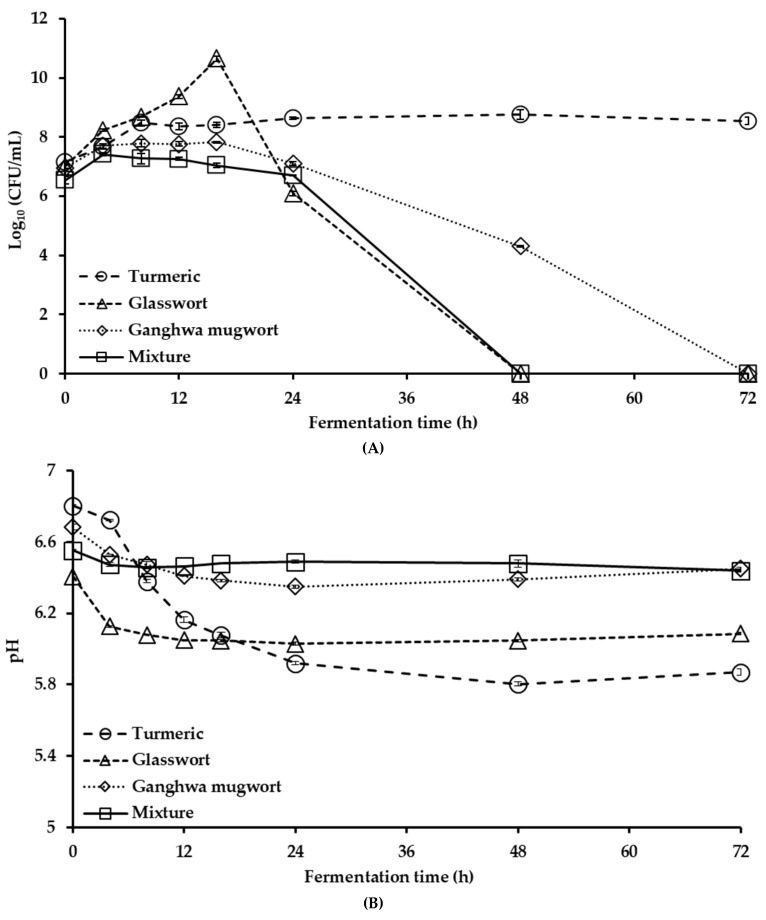
Changes in (**A**) bacterial cell counts and (**B**) pH during the fermentation of 5% (*w*/*v*) turmeric, 2.5% (*w*/*v*) glasswort, 2.5% (*w*/*v*) Ganghwa mugwort, and their 5% (*w*/*v*) mixture (1.66% (*w*/*v*) turmeric, 1.66% (*w*/*v*) glasswort and 1.66% (*w*/*v*) Ganghwa mugwort).

The fermentation profile of *E. faecium* varied with the medicinal plant used (Figure 1A). In turmeric fermentation, higher bacterial viability was observed at 8 h and maintained until 72 h. In glasswort and Ganghwa mugwort fermentation, the highest bacterial counts were observed at 16 h. For the mixture, it was observed at 4 h and maintained until 16 h. After 16 h of fermenting glasswort, Ganghwa mugwort, and their mixture, the viability of *E. faecium* gradually decreased. The pH was also decreased after 16 h of fermentation and remained virtually constant thereafter (Figure 1B). Based on the high bacterial counts and low pH, we selected 16 h for *E. faecium* fermentation.

### 3.4. Antioxidant Activities before and after Fermentation

**Figure 2:**
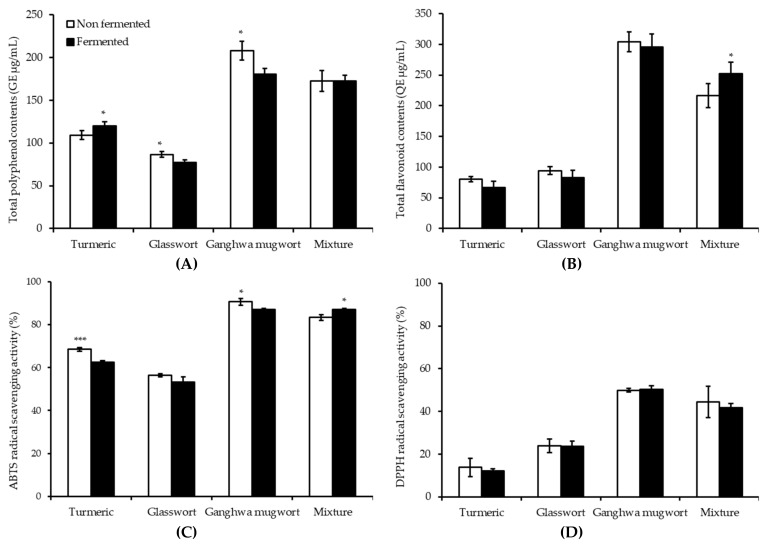
(**A**) Total polyphenol contents (gallic acid equivalent (GE) μg/mL), (**B**) total flavonoid contents (quercetin equivalent (QE) μg/mL), (**C**) 2,2’-azino-bis(3-ethylbenzothiazoline-6-sulfonic acid) (ABTS)_ radical scavenging activity, and (**D**) 1,1-diphenyl-2-picrylhydrazyl (DPPH) radical scavenging activity of the MeOH extracts of non-fermented and 16-h-fermented turmeric (5%, *w*/*v*), glasswort (2.5%, *w*/*v*), Ganghwa mugwort (2.5%, *w*/*v*), and their mixture (1.66% (*w*/*v*) turmeric, 1.66% (*w*/*v*) glasswort and 1.66% (*w*/*v*) Ganghwa mugwort). * *p* < 0.05, *** *p* < 0.001.

The TPC of medicinal plants displayed a mixed trend after 16 h of fermentation (Figure 2A). For instance, turmeric fermentation resulted in significantly increased TPC following fermentation (*p* < 0.05), while glasswort and Ganghwa mugwort showed a significantly decreased trend (*p* < 0.05). However, the mixture did not show any significant change in TPC following fermentation. Ganghwa mugwort showed the highest polyphenol content, whereas glasswort had the lowest TPC. The TFCs of all the medicinal plants were decreased after fermentation except the mixture, which showed significantly increased TFC after fermentation (*p* < 0.05) (Figure 2B).

The antioxidant capacities of samples before and after fermentation were measured based on their ABTS radical scavenging ability (Figure 2C) and DPPH radical scavenging ability (Figure 2D). The ABTS radical scavenging activity was the highest for Ganghwa mugwort. It was significantly decreased after fermentation (*p* < 0.05). The mixture after fermentation was shown to have the second-highest radical scavenging activity, which was significantly increased after fermentation (*p* < 0.05). Turmeric showed decreased radical scavenging activity with fermentation (*p* < 0.001). DPPH with free radical residue is a stable compound. It is commonly used to estimate an antioxidant as a substrate to measure proton-radical scavenging activity [32]. Antioxidant capacity analysis showed no significant difference in DPPH scavenging after *E. faecium* fermentation.

**Figure 3:**
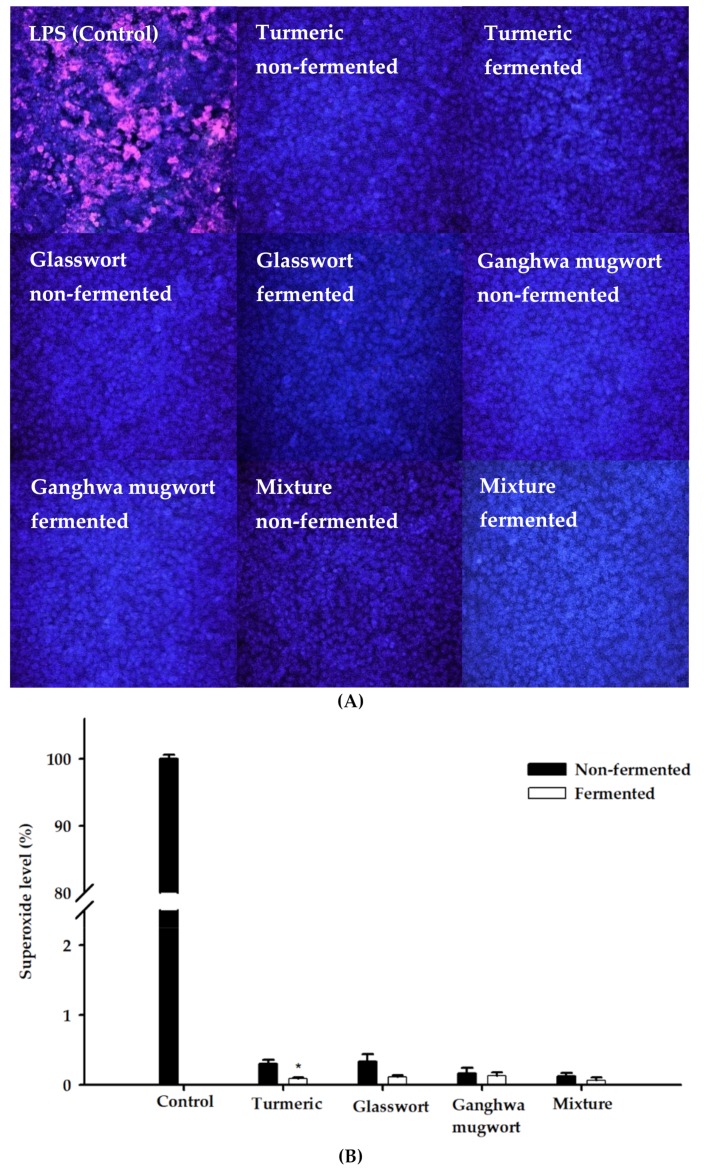
Antioxidant effects of fermented and non-fermented medicinal plants in Bovine mammary epithelial (MAC-T) cells. (**A**) Fluorescence microscopic image of lipopolysaccharide (LPS)-treated MAC-T cells. MAC-T cells were pre-treated with 50 μL/mL of fermented and non-fermented samples and DMSO (control) for 12 h, followed by exposure to LPS (1 μg/mL) for 4 h. Then, the cells were stained with dihydroethidium (DHE; superoxide probe) and mounted on cover slips with an antifade mountant containing DAPI to detect intracellular superoxide production. The cells were visualized with a fluorescence microscope. (**B**) Intracellular superoxide level as quantified by the fluorescence intensity and expressed as a percentage of control. Samples were non-fermented and 16-h-fermented turmeric (5%, *w*/*v*), glasswort (2.5%, *w*/*v*), Ganghwa mugwort (2.5%, *w*/*v*) and their mixture (1.66% (*w*/*v*) turmeric, 1.66% (*w*/*v*) glasswort and 1.66% (*w*/*v*) Ganghwa mugwort). * *p* < 0.05.

Comparative fluorescence microscopic images of control, non-fermented, and fermented samples of LPS-treated MAC-T cells are shown in Figure 3A. A superoxide anion can oxidize the fluorescent dye DHE, which stains cells a bright fluorescent red. The results showed that LPS (1 μg/mL) treatment of MAC-T cells increased superoxide production. Compared with LPS-treated MAC-T cells (control), cells after treatment with fermented and non-fermented medicinal plants showed almost no red color. This suggests a potent antioxidant effect against the LPS-induced oxidative stress of turmeric, glasswort, Ganghwa mugwort, and their mixture in MAC-T cells. The 16-h-fermented mixture dramatically changed the color of all cells and prevented the LPS-induced superoxide production. Superoxide level (%) was measured for MAC-T cells with or without any treatment by quantifying the fluorescence intensity (Figure 3B). Fermented samples quenched superoxide markedly higher as compared with the non-fermented samples. The lowest superoxide level was observed with the mixture treatment.

### 3.5. Antioxidant Activities of Dog Foods

**Figure 4:**
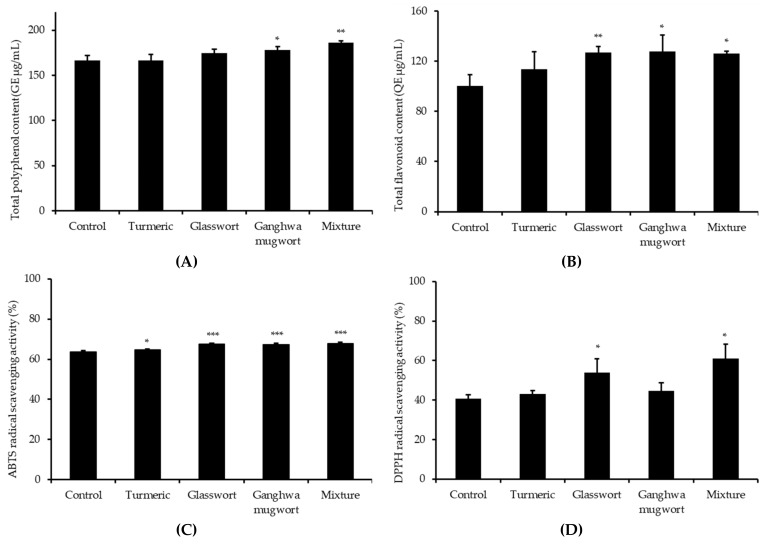
(**A**) Total polyphenol content (GE μg/mL), (**B**) Total flavonoid content (QE μg/mL), (**C**) ABTS radical scavenging activity, and (**D**) DPPH radical scavenging activity of dog foods. The samples were MeOH extracts with 1:3 ground dog food:80% MeOH. * *p* < 0.05, ** *p* < 0.01, *** *p* < 0.001.

TPC and TFC of dog foods between control and added with fermented medicinal plant are shown in Figure 4A,B. TPC of added Ganghwa mugwort and the mixture were significantly higher than the control (*p* < 0.05 and *p* < 0.01, respectively). Similarly, the TFC of Ganghwa mugwort and mixture were significantly higher than the control (*p* < 0.05). The TFC of glasswort was significantly higher than the control (*p* < 0.01).

The antioxidant capacities of samples before and after fermentation were measured based on ABTS radical scavenging ability (Figure 4C) and DPPH radical scavenging ability (Figure 4D). ABTS radical scavenging activities of all the treatments were significantly higher than the control. The DPPH scavenging capacities of dog foods with glasswort and mixture were significantly higher than the control.

### 3.6. Food Preference Test of Fermented Turmeric, Glasswort, Ganghwa Mugwort, and Their Mixture

The results of dog food preference using two-pan test are presented in Table 4. The beagles showed no resistance to eating foods supplemented with fermented turmeric, glasswort, Ganghwa mugwort, or their mixture. Although the beagles were similar in body weight during the study (data not shown), the food intake by individual beagles differed (Table 4). The intake ratios of the experimental dog food with fermented glasswort and Ganghwa mugwort were significantly higher than the control diet (*p* < 0.05). However, the addition of fermented turmeric decreased the acceptance of dog foods. Meanwhile, in the case of mixture vs. control diet, the mixture diet was consumed more, but not significantly so (*p* = 0.339).

### 3.7. Fecal Bacteria in Beagles Fed Turmeric, Glasswort, Ganghwa Mugwort, and Their Mixture

From the six feces samples obtained from beagles after food preference test, there were some overlapping dominant microbial species when control and treatment were compared (Table 5). The following seven species were isolated from the feces of control beagles: *Streptococcus lutetiensis*, *Acinetobacter baumannii*, *Myroides odoratimimus*, *Myroides odoratus*, *Lactobacillus acidophilus*, *Weissella paramesenteroides*, and *Lactobacillus animalis*. After 4 days of feeding on turmeric, dominant species were *Enterococcus alcedinis*, *Lactobacillus gasseri*, *L. animalis*, *L. acidophilus*, and *M. odoratimimus.* On the fourth day after feeding glasswort, dominant species were *Lysinibacillus pakistanensis*, *E. alcedinis*, *L. animalis*, and *L. gasseri.* Similarly, *E. alcedinis*, *L. animalis*, *L. pakistanensis*, *L. gasseri*, and *W. paramesenteroides* were dominant species after feeding Ganghwa mugwort. When the mixture was fed, *S. lutetiensis*, *L. animalis*, *L. gasseri*, and *Escherichia fergusonii* were dominant species. Overall, the number of pathogens such as *Streptococcus lutetiensis* decreased and the number of probiotics such as *Lactobacillus animalis* increased. When we fed dogs, foods treated with a fermented product, intestinal microbiota changed to a more beneficial composition.

## 4. Discussion

Consumers are beginning to demand dog foods improved by the addition of potential bioactive ingredients. In line with this trend, like the way people prefer foods that help delay aging, we have tried to include medicinal plants with excellent antioxidant abilities in dog foods. LAB fermentation is known to enhance the biological properties of plant matrices by providing lactic acid, probiotic LAB and increasing the bioavailability of polyphenols to the feeding animals, which may benefit host health. Lactic acid is used as a flavor, acidifying agent, and/or preservative in the food and feed industry [33]. Generally, probiotics for dogs and cats are used for gastrointestinal health and disease resistance [34]. Polyphenolic compounds are recognized to be health-promoting phytochemicals since they can act as antioxidants by radical scavenging [35]. Herein, the common fermentation strain *Enterococcus faecium* that we isolated and identified in each of the fermented materials was selected for study because *E. faecium* has been reported to have pH resistance, bile tolerance, heat resistance, and antimicrobial activity [36]. The isolated *E. faecium* strains also displayed safety profiles with respect to antibiotic resistance and hemolysis. 

In the present study, the increased TPC and TFC might have been due to the release of bound phenolics after fermentation while decreased TPC and TFC after fermentation highlighted the ability of *E. faecium* to metabolize these plants for their growth. The higher radical scavenging activity and intracellular antioxidant capacity after fermentation might be attributed to the production of some bioactive compounds after fermentation. These findings are in good agreement with an earlier report [37] which showed that during the fermentation of pomegranate juice, the concentration of phenolic compounds decreased whereas the antioxidant activity of fermented pomegranate juice increased. Another study has reported that the fermentation of turmeric powder significantly increased DPPH radical scavenging activity (*p* < 0.001) and ABTS cation radical scavenging activity (*p* < 0.01) [38]. Pianpumepong and Noomhorm [39] also showed increased TPC and antioxidant activities of turmeric following fermentation by autochthonous probiotic microorganisms (*E. faecium*, *Lactococcus lactis* subsp. *lactis*, and *Lactobacillus plantarum*). In our study, higher TPC, TFC, and antioxidant capacity of dog foods were recorded following the addition of fermented medicinal plants, which indicates the retention of plant metabolites (polyphenols) in the foods. Chen et al. [40] also reported the retention of tea polyphenols in dry dog foods. In a previous study, when glasswort was added at 2% to pork patties, antioxidant capacities were also increased [41]. However, the addition of phytogenic additives in dog foods might result in poor preference.

Few studies have been conducted to investigate dog food preference. It is noteworthy that food preference is one of the most important parameters to assess food acceptance among animals because the higher the food preference, the easier and more enjoyable is the administration of bioactive substances [40]. Intake ratios are the best indicators of overall food preference. An intake ratio greater than 0.50 represents a preference for a particular diet [42]. In the present study, except turmeric, all treatment diets were significantly preferred when dogs were offered a choice of a diet containing no fermented plants. Thus, these medicinal plant additives may present an odor that the dogs prefer, thereby increasing the food acceptance/preference. The decreased food preference with turmeric might be attributed to its strong flavor and/or bitter taste. For instance, dogs fed a high-fiber diet containing 8.5% sugarbeet pulp and 2% inulin showed lower voluntary food intake as compared with dogs fed a low-fiber diet containing 8.5% cellulose [43]. Even in the same material, preference changes depending on ingredients. A comparison of different concentrations of rapeseed meal revealed that intake ratio was high when organic matter, acid ether extract, and gross energy were low, but protein content was high in diet [44]. In the comparison of the preference of control food and two experimental foods containing 0.3% and 1% *Ascophyllum nodosum* for naïve dogs, the preference was significantly lowered in case of 1% addition to dog food [45]. Another study demonstrated that the addition of tea polyphenols (0.5%) in dry dog food could significantly increase the preference, antioxidant capacity, and antibacterial activity of dry dog food, while a decrease in intake ratio was observed as the concentration of tea polyphenols in the dog food reached 1.0% [40]. In the present study, the concentrations of different plants differed. Thus, preference results were affected by plants and their concentrations used. If the content of turmeric was reduced as for glasswort or Ganghwa mugwort, the food preference might have been improved.

Intestinal microbiota, also known as the gut microbiome, plays an important role in host health by modulating the immune system and development of gut structure in dogs [46]. Dog intestinal microbiota are greatly affected by the food consumed by the animal [47]. Several studies have used fecal samples as representatives of gut microbiota due to the ease of collection and non-invasiveness [48,49,50]. When dogs were supplied with a powder coating of *Bacillus* CIP 5832 to their dry food, *Bacillus* lived in the intestines, and was detected in feces. When it was not supplied continuously, *Bacillus* decreased in feces [51]. In addition, pig diet with added with herbal extract and organic acids appeared to reduce the proliferation of coliform bacteria [52]. In this study, fecal microbiota changed in a relatively good direction for groups fed with foods fermented by *E. faecium* as compared with the control group. For instance, *S. lutetiensis* isolated from the control group has pathogenic islands and virulence genes [53]. *A. baumannii* in the control group is an opportunistic nosocomial pathogen in human blood [54]. *M. odoratimimus* and *M. odoratus* are also low-grade opportunistic pathogens [55]. On the other hand, *L. acidophilus* and *L. animalis* are species used as probiotics [56,57] and *W. paramesenteroides* has probiotic characteristics [58]. *L. gasseri* also has probiotic characteristics [59,60]. Based on the results of other studies, it can be inferred that the changes in intestinal microbiota might be because of *E. faecium*, which is commonly used as a probiotic. *E. faecium* could significantly reduce the number of *Clostridium* spp. in feces as a result of oral feeding to 12 healthy house dogs [61]. In another study, diarrhea was significantly reduced in cats living in animal shelters by feeding *E. faecium* SF68 capsules [62]. Therefore, in the present study, the dietary addition of fermented medicinal plants could positively affect intestinal microbiota in dogs. However, further studies are required to validate this trend.

## 5. Conclusions

This study demonstrates that fermented glasswort and Ganghwa mugwort in dog food at 1% (*v*/*w*) inclusion led to good preference (*p* < 0.05) and displayed positive effects on the presence and function of intestinal microbiota. Moreover, the addition of fermented medicinal plants into dog foods showed no significant negative effects in food intake when compared to the control diet. These results suggest that diets supplemented with fermented turmeric, glasswort, Ganghwa mugwort, and their mixture, having enhanced antioxidant activities, could be used as nutritionally functional foods for dogs. However, future studies are imperative to understand the role of fermented medicinal plants in the antioxidant status of dogs.

## Figures and Tables

**Table 1:** Ingredients and chemical composition of experimental diets.

Item	Control	Treatment
Ingredient composition, as-fed basis (g/kg)		
Corn, grain	217.9	215.7
Chicken by-product meal	145.3	143.9
Corn gluten meal	79.9	79.1
Rice flour	72.6	71.9
Soybean meal	247.0	244.6
Beet pulp	7.3	7.2
Vitamin premix ^a^	10.9	10.8
Chicken fat	36.3	35.9
Premix ^b^	124.7	123.5
*Added by coating*		
Fermented medicinal plants ^c^	-	10.0
Salmon fat	50.8	50.3
Palatant enhancer ^d^	7.3	7.2
Nutrient composition, as-fed basis (%)		
Moisture	20.0	
Crude protein	24.0	
Crude fiber	10.0	
Crude fat	11.0	
Crude ash	15.0	
Calcium	0.2	
Phosphate	0.2	
Energy (kcal/100 g)	265.81	

The error range of nutrient composition was less than 1%. ^a^ Vitamin A, vitamin B, vitamin C, vitamin D, vitamin E, vitamin K, biotin. ^b^ Mn, Se, Mg, I, Co, lysine, DL-methion, tryptophan, threonine, choline, yucca extract, immunoprotein. ^c^ 1% added liquid samples and contents were as follows: 5% (*w*/*v*) turmeric, 2.5% (*w*/*v*) glasswort, 2.5% (*w*/*v*) Ganghwa mugwort, and 5% (*w*/*v*) mixture (1.66% (*w*/*v*) turmeric, 1.66% (*w*/*v*) glasswort, and 1.66% (*w*/*v*) Ganghwa mugwort). ^d^ Optimizor C2739.

**Table 2:** Isolation and identification of microorganisms from medicinal plants used in this study.

Material	Stock #	Description	Media	Coverage (%)	Identity (%)
Turmeric	SK4349	*Klebsiella pneumoniae*	NA	100	100
	SK4350	*Enterobacter cloacae*	NA	100	100
	SK4351	*Phytobacter diazotrophicus*	NA	100	100
	SK4352	*Klebsiella pneumoniae*	R2A	100	100
	SK4353	*Cronobacter sakazakii*	R2A	100	100
	SK4354	*Enterobacter aerogenes*	R2A	100	99
	SK4355	*Pediococcus pentosaceus*	MRS	100	100
	SK4356	*Enterococcus gallinarum*	MRS	100	100
	SK4357	*Enterococcus faecium*	MRS	100	100
Glasswort	SK4367	*Weissella cibaria*	MRS	99	99
	SK4368	*Enterococcus hirae*	MRS	100	99
	SK4369	*Enterococcus faecium*	MRS	100	100
	SK4370	*Enterococcus faecium*	NA	100	100
	SK4371	*Bacillus nealsonii*	R2A	100	99
	SK4372	*Enterococcus faecium*	R2A	100	99
Ganghwa mugwort	SK4373	*Enterococcus faecium*	MRS	100	100
	SK4374	*Weissella cibaria*	MRS	100	99
	SK4375	*Weissella cibaria*	MRS	99	99
	SK4376	*Enterococcus faecium*	R2A	100	100

**Table 3:** Antibiotic resistance of *E. faecium.*

Antibiotics	μg/disc	Susceptibility		
		SK4357	SK4369	SK4373
Cefepime	30	R ^a^	S ^b^	R
Gentamicin	2	R	S	S
Vancomycin	30	S	S	S
Ampicillin	10	S	S	S
Tetracycline	30	S	R	S
Oxacillin	1	R	S	R
Ciprofloxacin	5	S	S	S
Chloramphenicol	30	S	S	S
Clindamycin	2	R	S	S

^a^ Resistance of strain. ^b^ Sensitivity of strain.

**Table 4:** Food preference of experimental diets with and without fermented turmeric, glasswort, Ganghwa mugwort, and their mixture in beagles.

Item	Intake Ratio ^a^	
	Control	Treatment
Turmeric	0.54 ± 0.19	0.46 ± 0.19
Glasswort	0.40 ± 0.16	0.60 ± 0.16 *
Ganghwa mugwort	0.39 ± 0.15	0.61 ± 0.15 *
Mixture	0.48 ± 0.16	0.52 ± 0.16

^a^ Amount consumed of A/(amount consumed of A + amount consumed of B). Fermented medicinal plants were added to basal dog foods at 1% (*v*/*w*). * *p* < 0.05.

**Table 5:** Bacterial cell counts of beagle feces.

Diet with	LB		MRS	
	log_10_ (CFU/mL)	Species	log_10_ (CFU/mL)	Species
Control	8.9 ± 0.42	*Acinetobacter baumannii* *Myroides odoratimimus* *Myroides odoratus* *Streptococcus lutetiensis*	8.5 ± 0.63	*Lactobacillus acidophilus* *Lactobacillus animalis* *Streptococcus lutetiensis* *Weissella paramesenteroides*
Turmeric	9.1 ± 0.50	*Enterococcus alcedinis* *Lactobacillus animalis* *Myroides odoratimimus*	9.0 ± 0.50	*Lactobacillus acidophilus* *Lactobacillus animalis* *Lactobacillus gasseri*
Glasswort	8.8 ± 0.64	*Enterococcus alcedinis* *Lactobacillus animalis* *Lysinibacillus pakistanensis*	8.7 ± 0.58	*Lactobacillus animalis* *Lactobacillus gasseri*
Ganghwa mugwort	8.5 ± 0.59	*Enterococcus alcedinis* *Lysinibacillus pakistanensis*	7.4 ± 1.70	*Lactobacillus animalis* *Lactobacillus gasseri* *Weissella paramesenteroides*
Mixture	9.1 ± 0.75	*Escherichia fergusonii* *Lactobacillus animalis* *Streptococcus lutetiensis*	9.1 ± 0.75	*Lactobacillus animalis* *Lactobacillus gasseri* *Streptococcus lutetiensis*

The underlined strains are pathogenic.

## References

[1] Owens N., Grauerholz L. (2019). Interspecies Parenting: How Pet Parents Construct Their Roles. Humanit. Soc..

[2] McGee N., Radosevich J., Rawson N.E. (2014). Functional ingredients in the pet food industry: Regulatory considerations. Nutraceutical and Functional Food Regulations in the United States and Around the World.

[3] Di Cerbo A., Morales-Medina J.C., Palmieri B., Pezzuto F., Cocco R., Flores G., Iannitti T. (2017). Functional foods in pet nutrition: Focus on dogs and cats. Res. Vet. Sci..

[4] Hayek M.G. (2001). Process and product for enhancing immune response in companion animals using a combination of antioxidants. U.S. Patent.

[5] Sechi S., Chiavolelli F., Spissu N., Di Cerbo A., Canello S., Guidetti G., Fiore F., Cocco R. (2015). An antioxidant dietary supplement improves brain-derived neurotrophic factor levels in serum of aged dogs: Preliminary results. J. Vet. Med..

[6] Milgram N.W., Head E., Muggenburg B., Holowachuk D., Murphey H., Estrada J., Ikeda-Douglas C., Zicker S., Cotman C. (2002). Landmark discrimination learning in the dog: Effects of age, an antioxidant fortified food, and cognitive strategy. Neurosci. Biobehav. Rev..

[7] Bunghez F., Socaciu C., Catunescu G.M. (2012). Antioxidants used in pet feed. Bull. UASVM Agric..

[8] Glodde F., Gunal M., Kinsel M.E., AbuGhazaleh A. (2018). effects of natural antioxidants on the stability of omega-3 fatty acids in dog food. J. Vet. Res..

[9] Park C.Y., Lee K.-Y., Gul K., Rahman M.S., Kim A.-N., Chun J., Kim H.-J., Choi S.-G. (2019). Phenolics and antioxidant activity of aqueous turmeric extracts as affected by heating temperature and time. LWT.

[10] Kim J.H., Yang H.J., Kim Y.-J., Park S., Lee O.-H., Kim K.S., Kim M.J. (2016). Korean turmeric is effective for dyslipidemia in human intervention study. J. Ethn. Foods.

[11] Karthivashan G., Park S.-Y., Kweon M.-H., Kim J., Haque M.E., Cho D.-Y., Kim I.-S., Cho E.-A., Ganesan P., Choi D.-K. (2018). Ameliorative potential of desalted *Salicornia europaea* L. extract in multifaceted Alzheimer’s-like scopolamine-induced amnesic mice model. Sci. Rep..

[12] Patel S. (2016). Salicornia: Evaluating the halophytic extremophile as a food and a pharmaceutical candidate. 3 Biotech.

[13] Bae J.-Y., Park L.-Y., Lee S.-H. (2008). Effect of *Salicornia herbacea* L. powder on the quality characteristics of bread. J. Korean Soc. Food Sci. Nutr..

[14] Lim Y.-B., Kim H.-W., Hwang K.-E., Song D.-H., Kim Y.-J., Ham Y.-K., Jang S.-J., Lee C.-H., He F.-Y., Choi Y.-S. (2015). Effects of glasswort (*Salicornia herbacea* L.) hydrates on quality characteristics of reduced-salt, reduced-fat Frankfurters. Korean J. Food Sci. Anim. Resour..

[15] Han S., Kim S. (2003). Antioxidative effect of *Salicornia herbacea* L. grown in closed sea beach. J. Korean Soc. Food Sci. Nutr..

[16] Zhang J., Sasaki T., Li W., Nagata K., Higai K., Feng F., Wang J., Cheng M., Koike K. (2018). Identification of caffeoylquinic acid derivatives as natural protein tyrosine phosphatase 1B inhibitors from *Artemisia princeps*. Bioorg. Med. Chem. Lett..

[17] Hirano A., Goto M., Mitsui T., Hashimoto-Hachiya A., Tsuji G., Furue M. (2017). Antioxidant *Artemisia princeps* extract enhances the expression of filaggrin and loricrin via the AHR/OVOL1 pathway. Int. J. Mol. Sci..

[18] Carvalho I.S., Cavaco T., Brodelius M. (2011). Phenolic composition and antioxidant capacity of six Artemisia species. Ind. Crop. Prod..

[19] Bang M.-H., Cho J.-G., Song M.-C., Lee D.-Y., Han M.-W., Chung H.-G., Jeong T.-S., Lee K.-T., Choi M.-S., Baek N.-I. (2008). Development of biologically active compounds from edible plant sources XXII. Triterpenoids from the aerial parts of Sajabalssuk (*Artemisia princeps* PAMPANINI). Appl. Biol. Chem..

[20] Selhub E.M., Logan A.C., Bested A.C. (2014). Fermented foods, microbiota, and mental health: Ancient practice meets nutritional psychiatry. J. Physiol. Anthropol..

[21] Parvez S., Malik K.A., Ah Kang S., Kim H.Y. (2006). Probiotics and their fermented food products are beneficial for health. J. Appl. Microbiol..

[22] Cho S., Moon H.-I., Hong G.-E., Lee C.-H., Kim J.-M., Kim S.-K. (2014). Biodegradation of capsaicin by *Bacillus licheniformis* SK1230. J. Korean Soc. Appl. Biol. Chem..

[23] Foulquié Moreno M., Callewaert R., Devreese B., Van Beeumen J., De Vuyst L. (2003). Isolation and biochemical characterisation of enterocins produced by enterococci from different sources. J. Appl. Microbiol..

[24] Drew W.L., Barry A., O’Toole R., Sherris J.C. (1972). Reliability of the Kirby-Bauer disc diffusion method for detecting methicillin-resistant strains of *Staphylococcus aureus*. Appl. Environ. Microbiol..

[25] Dudonne S., Vitrac X., Coutiere P., Woillez M., Merillon J.M. (2009). Comparative study of antioxidant properties and total phenolic content of 30 plant extracts of industrial interest using DPPH, ABTS, FRAP, SOD, and ORAC assays. J. Agric. Food Chem..

[26] Lee O.H., Lee B.Y., Lee J., Lee H.B., Son J.Y., Park C.S., Shetty K., Kim Y.C. (2009). Assessment of phenolics-enriched extract and fractions of olive leaves and their antioxidant activities. Bioresour. Technol..

[27] Akowuah G., Ismail Z., Norhayati I., Sadikun A. (2005). The effects of different extraction solvents of varying polarities on polyphenols of *Orthosiphon stamineus* and evaluation of the free radical-scavenging activity. Food Chem..

[28] Jeong C.H., Ryu H., Zhang T., Lee C.H., Seo H.G., Han S.G. (2018). Green tea powder supplementation enhances fermentation and antioxidant activity of set-type yogurt. Food Sci. Biotechnol..

[29] Han S.G., Newsome B., Hennig B. (2013). Titanium dioxide nanoparticles increase inflammatory responses in vascular endothelial cells. Toxicology.

[30] Griffin R., Kvamme J., Phillips T. (2003). Palatability testing methods: Parameters and analyses that influence test conditions. Petfood Technol..

[31] Tôrres C.L., Hickenbottom S.J., Rogers Q.R. (2003). Palatability affects the percentage of metabolizable energy as protein selected by adult beagles. J. Nutr..

[32] Duh P.-D., Yen G.-C. (1997). Antioxidative activity of three herbal water extracts. Food Chem..

[33] Djukić-Vuković A.P., Mojović L.V., Semenčenko V.V., Radosavljević M.M., Pejin J.D., Kocić-Tanackov S.D. (2015). Effective valorisation of distillery stillage by integrated production of lactic acid and high quality feed. Food Res. Int..

[34] Grześkowiak Ł., Endo A., Beasley S., Salminen S. (2015). Microbiota and probiotics in canine and feline welfare. Anaerobe.

[35] Kayodé A.P., Mertz C., Guyot J.-P., Brat P., Mouquet-Rivier C. (2013). Fate of phytochemicals during malting and fermentation of type III tannin sorghum and impact on product biofunctionality. J. Agric. Food Chem..

[36] Park S.-M., Park H.-E., Lee W.-K. (2015). Selection and immunomodulatory evaluation of lactic acid bacteria suitable for use as canine probiotics. Korean J. Vet. Res..

[37] Mousavi Z.E., Mousavi S.M., Razavi S.H., Hadinejad M., Emam-Djomeh Z., Mirzapour M. (2013). Effect of fermentation of pomegranate juice by *Lactobacillus plantarum* and *Lactobacillus acidophilus* on the antioxidant activity and metabolism of sugars, organic acids and phenolic compounds. Food Biotechnol..

[38] Ra H.N., Kim H.Y. (2016). Antioxidant and antimicrobial activities of *Curcuma aromatica* Salisb. with and without fermentation. Korean J. Food Cook. Sci..

[39] Pianpumepong P., Noomhorm A. (2010). Isolation of probiotic bacteria from turmeric (*Curcuma longa* Linn.) and its application in enriched beverages. Int. J. Food Sci. Technol..

[40] Chen M., Chen X., Cheng W., Li Y., Ma J., Zhong F. (2016). Quantitative optimization and assessments of supplemented tea polyphenols in dry dog food considering palatability, levels of serum oxidative stress biomarkers and fecal pathogenic bacteria. RSC Adv..

[41] Joo S.Y., Choi H.Y. (2014). Antioxidant activity and quality characteristics of pork patties added with saltwort (*Salicornia herbacea* L.) powder. J. Korean Soc. Food Sci. Nutr..

[42] Trivedi N., Hutton J., Boone L. (2000). Useable data: How to translate the results derived from palatability testing. Petfood Ind..

[43] Farooqui A.A. (2015). Importance and roles of fiber in the diet. High Calorie Diet and the Human Brain.

[44] Kendall P.T., Holme D.W. (1982). Studies on the digestibility of soya bean products, cereals, cereal and plant by-products in diets of dogs. J. Sci. Food Agric..

[45] Isidori M., Rueca F., Trabalza-Marinucci M. (2019). Palatability of extruded dog diets supplemented with *Ascophyllum nodosum* L. (Fucaceae, Phaeophyceae). J. Appl. Phycol..

[46] Suchodolski J. (2011). Companion animals symposium: Microbes and gastrointestinal health of dogs and cats. J. Anim. Sci..

[47] Torrey J.C. (1919). The Regulation of the intestinal flora of dogs through diet. J. Med. Res..

[48] Mentula S., Harmoinen J., Heikkilä M., Westermarck E., Rautio M., Huovinen P., Könönen E. (2005). Comparison between cultured small-intestinal and fecal microbiotas in beagle dogs. Appl. Environ. Microbiol..

[49] Grønvold A.-M.R., L’Abée-Lund T.M., Sørum H., Skancke E., Yannarell A.C., Mackie R.I. (2009). Changes in fecal microbiota of healthy dogs administered amoxicillin. FEMS Microbiol. Ecol..

[50] Handl S., German A.J., Holden S.L., Dowd S.E., Steiner J.M., Heilmann R.M., Grant R.W., Swanson K.S., Suchodolski J.S. (2013). Faecal microbiota in lean and obese dogs. FEMS Microbiol. Ecol..

[51] Biourge V., Vallet C., Levesque A., Sergheraert R., Chevalier S., Roberton J.L. (1998). The use of probiotics in the diet of dogs. J. Nutr..

[52] Namkung H., Li J., Gong M., Yu H., Cottrill M., De Lange C. (2004). Impact of feeding blends of organic acids and herbal extracts on growth performance, gut microbiota and digestive function in newly weaned pigs. Can. J. Anim. Sci..

[53] Jin D., Chen C., Li L., Lu S., Li Z., Zhou Z., Jing H., Xu Y., Du P., Wang H. (2013). Dynamics of fecal microbial communities in children with diarrhea of unknown etiology and genomic analysis of associated *Streptococcus lutetiensis*. BMC Microbiol..

[54] Antunes L.C., Visca P., Towner K.J. (2014). *Acinetobacter baumannii*: Evolution of a global pathogen. Pathog. Dis..

[55] Maraki S., Sarchianaki E., Barbagadakis S. (2012). Myroides odoratimimus soft tissue infection in an immunocompetent child following a pig bite: Case report and literature review. Braz. J. Infect. Dis..

[56] Smit E., Oling F., Demel R., Martinez B., Pouwels P.H. (2001). The S-layer protein of *Lactobacillus acidophilus* ATCC 4356: Identification and characterisation of domains responsible for S-protein assembly and cell wall binding. J. Mol. Biol..

[57] Biagi G., Cipollini I., Pompei A., Zaghini G., Matteuzzi D. (2007). Effect of a *Lactobacillus animalis* strain on composition and metabolism of the intestinal microflora in adult dogs. Vet. Microbiol..

[58] Alvim L.B., Sandes S.H., Silva B.C., Steinberg R.S., Campos M.H., Acurcio L.B., Arantes R.M., Nicoli J.R., Neumann E., Nunes A.C. (2016). Weissella paramesenteroides WpK4 reduces gene expression of intestinal cytokines, and hepatic and splenic injuries in a murine model of typhoid fever. Benef. Microbes.

[59] Frolkova P., Svec P., Sedlacek I., Maslanova I., Cernohlavkova J., Ghosh A., Zurek L., Radimersky T., Literak I. (2013). *Enterococcus alcedinis* sp. nov., isolated from common kingfisher (*Alcedo atthis*). Int. J. Syst. Evol. Microbiol..

[60] Ventura M., Jankovic I., Walker D.C., Pridmore R.D., Zink R. (2002). Identification and characterization of novel surface proteins in *Lactobacillus johnsonii* and *Lactobacillus gasseri*. Appl. Env. Microbiol..

[61] Vahjen W., Manner K. (2003). The effect of a probiotic *Enterococcus faecium* product in diets of healthy dogs on bacteriological counts of *Salmonella* spp., *Campylobacter* spp. and *Clostridium* spp. in faeces. Arch. Anim. Nutr..

[62] Bybee S.N., Scorza A.V., Lappin M.R. (2011). Effect of the probiotic *Enterococcus faecium* SF68 on presence of diarrhea in cats and dogs housed in an animal shelter. J. Vet. Intern. Med..

